# A Systematic Review of Smartphone Applications That Address Patient Care in the Peri-Operative Period

**DOI:** 10.3390/healthcare13212775

**Published:** 2025-10-31

**Authors:** Hadal El-Hadi, Brandon Lok-Hang Chan, Brian Wai-Hei Siu, Ivan Ching-Ho Ko, David Ka-Wai Leung, Jeremy Yuen-Chun Teoh, Peter Ka-Fung Chiu, Chi-Fai Ng, Alex Qinyang Liu

**Affiliations:** 1 London School of Hygiene and Tropical Medicine, London WC1E 7HT, UK; 2 S.H. Ho Urology Centre, Department of Surgery, The Chinese University of Hong Kong, Hong Kongjeremyteoh@surgery.cuhk.edu.hk (J.Y.-C.T.); peterchiu@surgery.cuhk.edu.hk (P.K.-F.C.);; 3 Department of Urology, Medical University of Vienna, 1090 Vienna, Austria

**Keywords:** mobile health, digital health, surgery

## Abstract

**Background**: The use of smartphone applications by patients can be utilized to transform peri-operative care. With the ever-evolving landscape, an updated systematic review is needed in this field. **Objective**: This study aims to summarize the smartphone-based applications used by patients as discussed in the academic literature in the setting of peri-operative patient care. **Methods**: Seven databases were searched to identify articles discussing the use of smartphone applications by patients peri-operatively. Articles were included if they examined the use of smartphone-based applications in the setting of the peri-operative period and examined the application’s usability and effectiveness. Each paper was appraised using CASP checklists and analyzed using the thematic synthesis method. **Results**: Overall, 18 articles were selected for this study from 8204 articles initially obtained. The themes that emerged from the analysis include the following benefits of smartphone applications in peri-operative patient care: (1) patient education and instruction, (2) clear communication, (3) decreasing complications and the use of healthcare resources, (4) post-operative monitoring and pain control, (5) improved patient support, satisfaction, and safety. Other themes also emerged such as requirements of a practical smartphone application, what to include in smartphone application assessments, limitations of smartphone application studies, and future directions of smartphone applications regarding patient peri-operative care. **Conclusions**: The landscape of mobile applications is exponentially growing and their use in the peri-operative period is imminent for the future. Their use can improve communication between surgical care professionals, enhance patient care in the peri-operative period, and strengthen medical education. Further studies, validation tools, and improvements will be required to implement their use and demonstrate outcomes that can guide recommendations surrounding their use.

## 1. Introduction

The peri-operative surgical patient experience can be a complex series of clinical and administrative events that begins with the surgical referral and ends with the post-operative recovery process [1]. Surgical patient care can be implemented in an operating room, outpatient clinic, inpatient ward, emergency department, intensive care unit, or at home [2,3,4,5]. Smartphone and mobile health (mHealth) technologies are becoming integral to the evolution of telemedicine and digital health technology, as recent years have seen the increased adoption of smartphones by surgical care professionals and patients [6,7,8]. They also have the potential to optimize the quality of [7] and access to healthcare [8], enhance peri-operative patient support [9], reduce healthcare costs [10], support personal health management by encouraging healthy behaviour, improve adherence and self-management, and overall, change the face of surgery, patient care, and medical education [11,12].

The increased adoption of smartphones by surgical care professionals and patients across a wide age range makes it a logical tool that can improve communication and access to information systems and clinical tools at the point of care [1,8]. New advances in smartphone applications (apps) provide opportunities for innovative patient care, real-time data delivery, and patient–provider engagement from anywhere at any time [7,8]. Standardizing patient care by utilizing smartphone apps can address key components in access to care, patient safety, and healthcare quality by eliminating variations in patient education, recovery monitoring, treatment compliance, counselling, and accessibility to providers [1,13].

There is an emerging interest in smartphone apps by surgical care providers with an increased focus on patient-centred care. Numerous apps are available that can assist in the peri-operative period [8]. Patient education plays an important role in the treatment process, with the aim of developing self-management skills to facilitate recovery and ensure long-term success [14]. Apps can help patients enhance their involvement in their management, for example, by providing an opportunity to enter their own data and symptoms on their smartphone that can be relayed in real time to the care team, who can then act on abnormal trends in symptoms [6,8,14]. Apps can be used not only as a technology adjunct for point-of-care and as a diagnostic tool but also to send medication and appointment reminders [6,8,15]. Apps must address best practices and regulatory standards with regard to privacy, security, and patient confidentiality [6].

In this systematic review, the peri-operative period is defined as the time between the beginning of the pre-operative period (the period between the decision for operation until the operation) and the end of the post-operative period (the period where a patient is being followed up for his/her post-operative recovery). The current mobile health landscape for surgical patients is heterogeneous, and many different efforts have been made to incorporate technology with peri-operative care. However, there has been a gap in the literature to summarize the evidence supporting their use by patients. There have been small-scale randomized control trials (RCTs) to assess the use of apps, with outcomes such as patient-rated satisfaction, anxiety, patient awareness, behavioural change, and complication detection. However, no literature review currently summarizes the findings, specifically in the peri-operative setting. As there is an emerging interest in apps by surgical care providers, and as more apps are developed to help patients in the peri-operative period, completing this study will be of interest and relevance to all surgical care providers and patients who currently are interested in the use or development of patient care peri-operative smartphone apps.

### Aims and Objectives

This project describes recent clinically tested apps in the scientific literature and how they were integrated into peri-operative patient care. It aims to describe the current scope of app use within the peri-operative period for patients, classify and summarize how they are used according to their functionalities and benefits, and finally, review the outcomes of their use. Apps found in the recent scientific literature were classified into areas of benefits that were deduced from the analysis of the included studies. Limitations of these studies, as well as future directions of smartphone apps, were also examined. Therefore, this review aims to fill in the gap in the literature by meeting the following objectives:

1. To describe the current attempts in smartphone apps to aid in the peri-operative care of patients by identifying and classifying published information in the literature on their benefits and perceived outcomes.
2. To identify and summarize effective smartphone app features, assess the usability and effectiveness of the apps to help guide future studies, identify the limitations of the studies included in the project, identify gaps in the literature, and identify areas of interest for further clinical studies.

## 2. Methodology

### 2.1. Research Question

The research question was created by defining and developing a specific searchable question from a research topic. The research question was developed according to the Sample, Phenomenon of Interest, Design, Evaluation, Research type (SPIDER) tool derived from the Population, Intervention, Comparator, Outcome (PICO) tool. For the research question “how are smartphone apps utilized in the peri-operative period by surgical patients, what mobile applications have been described in the published literature and what are their benefits, utility, features, limitations and future directions in peri-operative patient care?”, the SPIDER framework was utilized as follows:

(S)ample: Surgical patients.

(P)henomenon of (I)nterest: Use of smartphone applications in the peri-operative period that involves patient care.

(D)esign: Structured literature review using thematic synthesis.

(E)valuation: Smartphone application benefits, utility, features, limitations, and future directions.

(R)esearch type: Qualitative.

### 2.2. Search Strategy

The research question was then used to create searchable concepts. In June 2022, seven appropriate databases (MEDLINE/Pubmed, Embase, Academic Search Complete, BASE, Cochrane Library, International HTA database, and Open Grey) were searched to identify articles that discussed the use of smartphone applications by surgical patients in the peri-operative period. Relevant search terms were identified and chosen to be included in a search strategy, including free text terms (keywords) and subject headings. Prior to a complete search, a discussion with a senior librarian from the London School of Hygiene and Tropical Medicine (LSHTM) helped guide the process through advice regarding search terms and databases. Search term concepts that discuss the design, development, evaluation, or use of smartphone applications to be used by surgical patients around patient care helped inform the MeSH vocabulary and included “mobile application”, “peri-operative”, and “patient care”, with each term further incorporating relevant synonyms and alternative terms. An effective search strategy was constructed by compiling and connecting the words and phrases together, making appropriate use of common search techniques such as Boolean operators and truncation. Filters were used to exclude non-English papers, and no limit was selected regarding the year of publication or status. An example of the search strategy is provided as follows:

(mobile app*) OR (phone app*) OR (web app*) OR (online app*) OR (iPhone app*) OR (smartphone app*) OR (mHealth) OR (application software*) OR (software app*) OR (app software*) OR (android ap*) OR (cellular app*) OR (cellphone app*) OR (cellular phone app*) OR (health care app*)

AND

(periop*) OR (peri-op*) OR (surgery) OR (surgical) OR (pre-op*) OR (preop*) OR (postop*) OR (post-op*) OR (intraop*) OR (intra-op*) OR (prehospital) OR (pre-hospital) OR (posthospital) OR (post-hospital) OR (inhospital) OR (in-hospital) OR (pre-surg*) OR (presurg*) OR (postsurg*) OR (post-surg*).

### 2.3. Inclusion Criteria

1. Full text.
2. English language.
3. Primary research.
4. Used by patients who underwent any type of surgery.
5. Interventions aiming to improve post-operative care.
6. Interventions using mHealth.
7. Patient care-oriented.
8. Focused on the design, development, evaluation, or use of a smartphone-based app.
9. Discussed phone apps that had the ability to function independently without the need for medical device.
10. Discussed the features and utility of the app.

### 2.4. Exclusion Criteria

1. Did not meet the inclusion criteria listed above.
2. Studies not in English.
3. Unavailable full-text articles.
4. Studies that were research protocols, conference presentations, reviews, editorials, case reports, and case series.
5. Smartphone technology used for health intervention as opposed to patient care were excluded.
6. Smartphone apps whose target audience were surgical care providers as opposed to patients.
7. Did not discuss the features and utility of the app.

### 2.5. Study Screening, Selection, Analysis, and Quality Appraisal

For assessing the methodological quality of the included studies, four major areas were assessed, including the design, features, benefits, and analysis of the studies. For the selection process, the Preferred Reporting Items for Systematic Reviews and Meta-Analyses (PRISMA) guidelines for systematic reviews were adhered to [16]. The information created was carefully managed during the search process. Selected articles were exported using the Endnote referencing manager and the results of all database searches were imported to Mendeley and manually de-duplicated. Titles were screened for relevance, and those that fell within the scope of the project were screened by abstract to identify relevant papers. The titles and abstracts screened had to satisfy the inclusion criteria described above, and those that did not were excluded. Full texts of articles located during the literature search were accessed. The full text of these papers was then examined to determine eligibility for the final studies. An in-depth review was then conducted, and qualitative data was extracted. The reference lists of included articles were also searched systematically and assessed for eligibility in a snowballing process. Quantitative studies were assessed by the STrengthening the Reporting of OBservational Studies in Epidemiology (STROBE) guidelines [17] and qualitative studies were assessed by the Qualitative Research Review Guidelines (RATS) guidelines [18]. While extracting data from the included studies, several themes began to emerge, including (1) patient education and instruction, (2) clear communication, (3) decreasing complications and the use of healthcare resources, (4) post-operative monitoring and pain control, (5) improved patient support, satisfaction, and safety. Other themes also emerged, such as the requirements for an effective smartphone application, what to include in smartphone application assessments, limitations of smartphone application studies, and future directions for smartphone applications with regard to patient peri-operative care. The data was narratively summarized in a concise fashion using a thematic synthesis.

### 2.6. Ethics Approval

This study was approved by LSHTM’s ethics committee (reference number: 27003) on 5 May 2022. As this project is a systematic/literature review only, it was assessed by the Research Governance and Integrity Office as not requiring ethical approval from the ethics committee. This study was also conducted according to universal ethical principles. This study was not registered publicly otherwise.

## 3. Main Report—Results

### 3.1. Search Results

**Figure 1:**
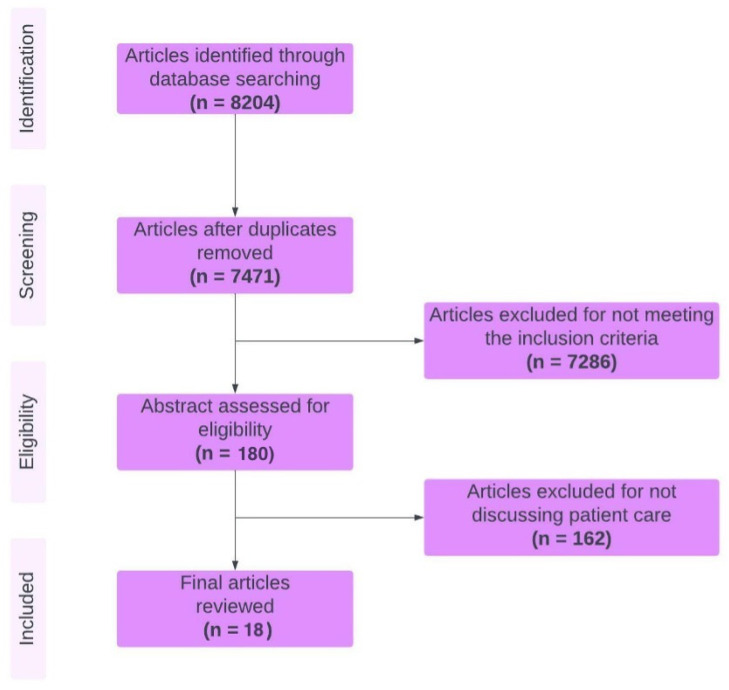
PRISMA flowchart of study selection.

The literature search resulted in 8204 articles from seven databases. Duplicate articles, of which there were 733, were removed, and the inclusion and exclusion criteria described above were applied, resulting in the exclusion of 7286 articles. The remaining 185 articles were initially screened based on the titles and abstracts. These 185 were reviewed in full text, and an additional 162 were excluded as they did not describe the perceived outcome of the phone application regarding patient care. Twenty-three studies were selected for an in-depth review as they met the inclusion criteria. A flowchart of eligible articles for this project is shown in Figure 1.

### 3.2. General Description of the Included Studies

Twenty-three articles that described the current scope of mobile application use within the peri-operative period for surgical care providers and their patients were analyzed. Table 1 describes the paper, the application, its utility, and the perceived outcomes.

### 3.3. Thematic Synthesis

**Figure 2:**
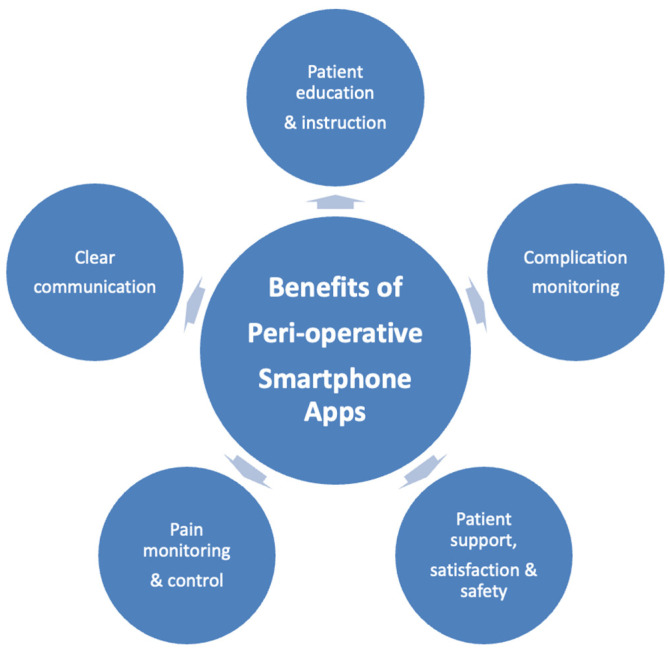
Five key benefits of peri-operative patient care smartphone applications.

The themes that emerged from the analysis include the benefits of smartphone applications in peri-operative patient care, which includes five aspects that are summarized in Figure 2: (1) patient education and instruction, (2) clear communication, (3) complication monitoring, (4) pain monitoring and control, (5) improved patient support, satisfaction, and safety. Other themes also emerged such as requirements of an effective smartphone application, what to include in smartphone application assessments, limitations of smartphone application studies, and future directions of smartphone applications regarding patient peri-operative care.

## 4. Discussion

The findings of this systematic review highlighted several recurring domains in which smartphone applications have been applied to peri-operative patient care. To provide clarity and coherence, the discussion is structured around the aforementioned domains, which reflect both the most frequently reported functions and the outcomes most relevant to surgical patients and their providers. These categories capture the breadth of potential benefits described in the literature, align with established priorities in peri-operative care, and offer insight into how digital health interventions may address key challenges faced by patients and healthcare systems.

### 4.1. Patient Education and Instruction

Effective patient education and instruction empower patients to actively participate in their own recovery process peri-operatively, and has been linked directly to improved satisfaction, reduced anxiety, the decreased utilization of the emergency department for surgery-related concerns, decreased readmission rates, and decreased overall health costs [1,15,35,36,37,38,39]. Smartphone applications, in particular, provide specific information via a highly accessible format [40,41]. Mobile technology improves surgical education and instruction throughout the pre-operative and post-operative timeframes and data towards the acceptance of the use of mobile technology for peri-operative education appears favourable [1]. Pre-operative education improves the patients’ post-operative coping abilities, and mobile applications emerge as an important tool to engage patients in pre-operative education about their condition and procedure [42].

A significant concern regarding patient-facing mobile applications is the potential for outdated or inaccurate content [40]. Ensuring the accuracy and relevance of such information requires direct input from surgical care providers, including peri-operative nurses and surgeons [1]. It is imperative that the information provided through these applications remains consistent with the guidance offered by surgical care providers during counselling sessions to prevent contradictions and maintain patient trust. When digital applications deliver reliable and precise information, they complement in-person pre-operative counselling, thereby enhancing the overall surgical experience for patients.

### 4.2. Clear Communication

Effective and precise communication throughout the peri-operative period is essential for delivering high-quality surgical care. Nevertheless, patients frequently misinterpret or fail to retain conveyed information during surgical consultations. For example, prior studies on the ability of patients to recall information, immediately after counselling after their pre-operative visit, demonstrated poor recall of procedural risks as explained in the informed consent process [43]. This issue is exacerbated by the widespread availability of online resources, with varying quality that may not be tailored to specific surgical procedures, potentially causing confusion for patients seeking accurate information [1].

mHealth applications have the benefit of providing specific educational information on the surgery itself and the peri-operative process, on top of conventional peri-operative counselling. A randomized controlled trial evaluated a mobile application providing information about the peri-operative period by text and video animations, and recovery advice based on a personalized convalescence plan, compared with a control of standard patient information leaflets [33]. The findings demonstrated a statistically significant reduction in the median time to resume normal activities. Numerous other mHealth applications offer on-demand multimedia resources to patients, aiming to improve information retention beyond clinical settings [1,9,19,28,30]. As such, mHealth applications have the potential to offer substantial benefits, given the limited retention of medical details during consultations; these tools may deliver procedure-specific, standardized content that bolsters information recall, promotes adherence to health directives irrespective of the time or location, and reduces discrepancies in guidance across healthcare providers [43,44,45,46,47,48].

### 4.3. Decreasing Complications and Use of Healthcare Resources

The first 30 days after an operation is critical, given that most complications occur during that period [2,3]. Efforts to improve patient education and peri-operative experiences have decreased the length of stay (LOS), readmission rates, and emergency department visits, enhancing cost-effectiveness and increasing healthcare service efficiency [1,9,48,49]. Non-optimal post-operative care can lead to preventable complications such as long-term disability, chronic pain, organ rejection, and death [50]. Smartphone applications can inform patients about possible complications and potential warning signs contributing to preventable morbidity and mortality [11]. Smartphone applications can also remotely monitor patient recovery, improve the completion of post-operative follow-up appointments, monitor non-severe complications, and provide surveillance of wound infections, which decreases unscheduled visits [51,52,53,54,55,56,57]. However, studies examining the role of mHealth applications in reducing post-operative complications or LOS were largely limited to single-arm studies or retrospective cohort studies [28,29,32]. While the included studies were invaluable for establishing the feasibility and user acceptance of mHealth applications, randomized controlled trials may be necessary to conclusively establish the causal efficacy of applications in improving clinical outcomes.

### 4.4. Post-Operative Monitoring and Pain Control

In surgical care, remote monitoring may allow improved transition care and greater insight regarding the development of post-operative complications and readmissions [6]. Mobile apps facilitate the collection of patient-reported outcomes (PROs) on potentially concerning post-operative symptoms, such as pain, headache, weakness, and vomiting. In one example, if any of the symptoms were reported, the patient could be directed to education resources tailored to their condition(s), with reported data available for review by the anesthesiologist and/or surgeon [19]. In another study, orthopedics patients reported their post-operative pain control and opioid use via a messaging-based application, with a notably high 96.1% response rate [21], demonstrating the feasibility and efficacy of mobile applications as a robust alternative to traditional methods for collecting PROs. In addition to reporting outcomes, patients can initiate contact with healthcare providers, for concerns such as pain and wound issues, highlighting possibilities for two-way communication enabled by smartphone applications [58].

Instructions regarding post-operative pain medication use can potentially decrease opioid requirements, and implementing pain measurements and content on how to reduce pain medication could reinforce a positive attitude of users toward the application [1]. Research shows that the usage of interactive systems, chat interactions, videoconferencing sessions, and phone counselling improves physical function, disability, and pain compared with conventional methods of information delivery [59]. Pain disability, in particular, is largely influenced by how patients interpret and adapt to their pain [60]. As such, mobile applications represent not only a monitoring technique for post-operative pain, but also a promising approach to guide pain control, and to reduce pain medication intake [1].

One important caveat is the high heterogeneity in post-operative patient-reported outcomes (PROs) and pain score measures across the mHealth landscape. Additionally, many reported studies have inherent methodological limitations of single-arm designs. More studies with randomized controls are likely necessary to demonstrate the potential benefits of mHealth applications in post-operative pain control. For instance, a randomized controlled trial evaluating an intervention based on Acceptance and Commitment Therapy (ACT) delivered via text messages reported reduced opioid use [20]. Thus, mobile applications with integrated post-operative pain monitoring and tailored pain management strategies may offer a new avenue for enhancing patient outcomes and reducing the reliance on opioids post-surgery, but higher-quality evidence from well-designed studies is essential to substantiate these findings.

### 4.5. Improved Patient Support, Satisfaction, and Safety

Comprehensive care for surgical patients encompasses not only the intra-operative phase but also the pre-operative and post-operative periods, which are equally vital for optimizing outcomes. Effective patient support and communication during these stages play an important role in enhancing both patient satisfaction and safety [1]. Smartphone applications have reported advantages for a patient’s sense of being looked after; robust communication ensures patients are well informed about wound care, activity restrictions, and potential complications, providing reassurance and empowering them to actively participate in their recovery process [40,61]. Patients who are highly engaged in self-management derive more benefit from using mHealth apps than others [40]. Furthermore, mHealth applications offer a secure, accessible platform for two-way communication with clinicians, extending patient–provider interactions beyond the consultation room and fostering greater patient engagement in the recovery process [40,62].

### 4.6. Requirements of an Effective Smartphone Application

For high-yield benefit and impact for a smartphone application, several critical features should be included (1). These features are important when considering the wide age range of patients and the diverse range of technological literacy among patients [1]. To increase the relevance of app use, it is preferable that mHealth apps include diverse functions that enable patients to personalize and tailor them to meet their needs [40,41]. The pre-requisites of an ideal smartphone application are summarized in Table 2.

### 4.7. What to Include in Smartphone Application Assessments

Key considerations for evaluating the success of a mobile health (mHealth) application encompass its usability, user experience, and the knowledge acquisition facilitated by the tool. Usability may be assessed through established instruments, such as the System Usability Scale (SUS), originally devised by John Brooke in 1986, which has since been validated across diverse technological domains as a robust indicator of effectiveness, efficiency, and overall satisfaction [62,63]. The SUS comprises a 10-item questionnaire employing a 5-point Likert scale, ranging from 1 (strongly disagree) to 5 (strongly agree).

Furthermore, users’ specific attitudes toward the application can be quantified using validated tools like the eHealth Impact Questionnaire (eHIQ), which features three subscales: (1) confidence and identification (nine items), (2) information and presentation (eight items), (3) understanding and motivation (nine items) [64]. This validated instrument evaluates the experience encountered by users during interactions with health-oriented digital platforms.

User outcomes could likewise be appraised via attitude-oriented questionnaires, incorporating formats such as numeric rating scales, 5-point Likert scales, multiple-choice items, and open-ended responses to enable both descriptive statistical analyses and inductive thematic content analyses. Table 3 delineates the pertinent data metrics to integrate within these user questionnaires.

The most important benchmark for mHealth applications is patient engagement and user uptake. Monitoring the number of total visits to the mobile application per participant is essential for evaluating the application’s usability. Key user behavioural metrics for further development and implementation include the total number of visits, the timing of visits during the day, and the duration of each interaction [19].

### 4.8. Limitations of Smartphone Application Studies

The articles included were all in English, and smartphone application usage may differ globally between English-speaking countries and other geographical specifications. Furthermore, the exclusion criteria of most of the studies excluded several patient demographics that are different from the reality of the diverse patient population that surgical care providers regularly encounter, such as patients with cognitive impairments, mental illnesses, or limited language skills. Most of the articles studied smartphone applications that were developed and provided in English, and language limitations would have biassed the studies. The studies were also completed in urban tertiary academic centres. Though many of the apps were specifically developed to meet the needs of their target group, patient technology literacy cannot be entirely eliminated, thereby, as a further limitation, negatively affecting patient adaptation and compliance to smartphone application utilization [15].

Most of the articles studied applications only available on the iOS App Store and iOS platforms, which meant that participants had to own an iOS smartphone with internet access leading to a significant selection bias. Some apps were also available on Android platforms, but that was less common. Most of the studies excluded patients with any self-reported condition that impaired their ability to use mobile applications, answer questionnaires and surveys, and/or provide insights representative of the general population, such as blindness or the active influence of recreational drugs or alcohol. A major limitation of most of the studies includes platform engagement being more likely in participants who signed up for a study [19].

All of the above limitations raise concerns about selection bias, as the patients included may disproportionately represent English-speaking, better educated, and higher income participants, reducing the external validity and generalizability of findings. Future studies should therefore increase the inclusivity to further reduce bias.

The majority of the articles included in this study used surveys to extract data regarding the perceived outcome of the smartphone application being discussed. Surveys commonly have low response rates with small sample sizes, and most papers were only able to collect de-identified data. Due to this, it is difficult to elicit which patients benefited the most from using smartphone applications. There is also a wide variation in how counselling is provided to patients based on surgeon preference. Given this, there may be subtle differences between in-office counselling from specific surgeons and the standardized information sent to patients [1].

All the articles included in this study were based on patients who were over the age of 18. Information was not stratified based on age, and some studies showed that middle-aged and older users pay more attention to their health issues and are more motivated to take action by using mHealth to avoid illness and stay healthy [65]. However, other studies have shown that younger, higher-educated, and higher-income patients were more likely to use smartphone applications. This variation in how age groups utilize smartphone applications shows a gap in the research, as different age groups may have different experiences of app usability and other expectations for how apps should function [65]. There are also no studies to provide insight into the use of mHealth amongst younger patients who may be more confident in their post-operative recovery course, less motivated to manage their health, and less focused on specific health management. None of the articles used semi-structured interviews to help to define areas that could be further explored about smartphone application usage and could have given more detailed information about some themes [66].

Telehealth has taken an ever-increasing role in patients’ access to healthcare, particularly in the context of physical distancing and resource limitations presented during the coronavirus (COVID-19) pandemic [67]. Smartphone apps enable remote consultations, health monitoring, and patient education, thereby minimizing the need for in-person visits that could increase exposure to infectious diseases [19]. Future directions for smartphone apps would be to address the issues related to their confidentiality, staffing, time requirements, and medical–legal issues that can facilitate effective physician–patient contact through a patient care app [68]. Moreover, developing and deploying multiple peri-operative mobile applications within the same institution or same department may be confusing to both patients and clinicians. Coordinated efforts across departments or institutions are needed to standardize the functionalities and workflows of peri-operative mHealth applications and to ensure seamless continuity of care [19].

A major concern for the deployment of mHealth applications remains the barriers to access for diverse individuals, especially those across the socioeconomic spectrum [69,70,71,72,73]. A fundamental principle for peri-operative mHealth applications should be to minimize costs to the patient and ensure equitable access [6]. While some patients may not have access to smartphones, or their complex needs may not be well addressed with mHealth applications, the implementation of these applications can enable the more efficient allocation of resources to support these individuals [19].

Smartphone apps and patient care interventions are multifactorial in nature. Future research can elucidate which component(s) or feature of a smartphone app makes the most difference in outcomes and which are the most cost-effective [19]. Future research should also focus on differences in app usability and utility amongst diverse demographics, such as age groups, gender, racial and other minoritized patients, and patients with disabilities [19]. Future studies should also include the introduction of the app in the early pre-operative period, interdisciplinary collaboration to expand the scope and quality of the app, pilots in larger and more diverse populations, interaction among healthcare workers and patients within the app, and the integration of quality of recovery scores [74].

When assessing the applications included in this review against the idealized features summarized in Table 2, most incorporated only a subset of functionalities, such as peri-operative education, reminders, or symptom monitoring. Furthermore, the extent to which these features translated into measurable improvements was inconsistently reported. Only a minority of studies explicitly reported that the app content was developed or validated by clinical experts such as surgeons, anesthesiologists, or peri-operative nurses. Even fewer described mechanisms for the dynamic or regular updating of educational material. This lack of transparency raises concerns about the risk of outdated or inconsistent information being delivered to patients, which may undermine patient trust or contribute to misinformation. As patient-facing mHealth tools continue to expand, future app development and study should ensure the formal involvement of clinical specialists in content design and incorporate processes for timely updates, with validation studies performed. Such measures are critical to safeguard the accuracy, reliability, and long-term value of peri-operative smartphone applications.

Although this systematic review employed a rigorous search strategy across seven databases, only 18 studies ultimately met the inclusion criteria. This sharp reduction reflects the scarcity of high-quality, patient-facing peri-operative mobile-health studies despite the large and expanding field of digital health. As a result, several important domains remain under-represented. For instance, few studies evaluated long-term outcomes beyond the immediate post-operative period, cost-effectiveness, or the integration of artificial intelligence and data analytics for personalized care. Likewise, research on app implementation in resource-limited settings, non-English-speaking populations, and pediatric or geriatric cohorts was minimal. Future work should therefore expand to these neglected areas and adopt standardized reporting frameworks, mixed-methods designs, and multicentre randomized trials to establish stronger, generalizable evidence for peri-operative mHealth interventions.

Lastly, a key limitation of this review is that the literature search was conducted up to 2023, and therefore more recent publications (2024 onwards) are not included in the synthesis. While we recognize that new studies may have since emerged that could enrich the evidence base, the present review remains valuable by providing the first comprehensive synthesis of data available up to 2023. We believe that this still establishes an important reference point for the field. Future work should aim to incorporate the most recent evidence and assess whether newer findings alter or reinforce the conclusions presented here.

## 5. Conclusions

This systematic review highlights the growing role of smartphone applications in peri-operative care. They are used to connect patients and healthcare teams and to educate patients, encourage disease self-management, and support the remote monitoring of symptoms. The provision of timely information and instructions via smartphones has the potential to enhance patients’ knowledge, comprehension, adherence to medication or treatment regimens, satisfaction, and clinical outcomes. The included studies consistently demonstrated benefits in patient education, clearer communication, complication monitoring, post-operative pain control, and enhanced patient support and satisfaction. Together, these findings suggest that mHealth applications can empower patients, improve adherence, and strengthen communication with surgical teams. At the same time, limitations remain, including small sample sizes, methodological heterogeneity, and potential selection bias, underscoring the need for more rigorous and inclusive research. Future work should prioritize randomized controlled trials, diverse patient populations, and standardized outcome measures to better validate effectiveness.

In conclusion, in the era of patient-centred care, mHealth apps play a vital role in improving patient care through improving communication between the surgical providers and patients, thus improving patient satisfaction. This systematic review found that using a mobile application to provide patients with educational material or instruction is feasible and beneficial. Furthermore, the collection of data on patient engagement and the evaluation of these applications informs the development of more effective, tailored peri-operative tools. This project contributes to the growing body of evidence supporting the utility of telehealth strategies in the peri-operative period, a demanding time in patients’ lives.

## Figures and Tables

**Table 1:** Description of included studies.

Study	Year	Country	Study Design	Specialty (Procedures Included)	Sample Size (n)	Name of Application	Themes of Application Identified	Specific Features	Format	Outcome Measures	Study Outcomes	SUS
Morte et al. [1]	2021	United States	Single-arm study	General surgery (elective cholecystectomy, interval appendectomy, colectomy, bariatric surgery, and open hernia repairs)	100	MCare	Patient education and instruction	Peri-operative educational materials; text message reminders of time-sensitive events	Text messages	- Usability	- 86% of patients felt that the application improved their surgical experience	- Average SUS of 86 (>90th percentile)
Williems et al. [9]	2021	The Netherlands	Cross-sectional study	Orthopedics (surgery for musculoskeletal disorders)	526	Patient Journey	Patient education and instruction	Educational materials on pre-operative exercises, surgery, and rehabilitation	iOS and Android apps	- Usability - User attitude - User experience	- Positive attitude toward information provided via app (eHIQ median 78) - Did not improve users’ confidence in discussing health with others or motivation to manage health	- Median SUS of 85
Ke et al. [19]	2021	Canada	Single-arm study	Obstetrics (cesarean delivery)	36	C-Care	Complication monitoring; pain monitoring and control; patient education and instruction	Education materials on peri-operative anesthetic topics; self-monitoring questionnaire on pain and complications	iOS app	- User engagement - User satisfaction	- Median of 3 out of 5 self-monitoring questionnaires completed per user - Median of 15 application visits over 30 days per user - Median patient satisfaction was 7.5 out of 10	N/A
Anthony et al. [20]	2020	United States	RCT	Orthopedics (operative fixation of a traumatic upper or lower fracture)	82	N/A	Pain monitoring and control	Acceptance and Commitment Therapy delivered through text messages	Text messages	- Amount of opioid pain medication consumed - Changes in patient-reported pain scores, measured by PROMIS	- 36.5% less opioid tablets used by intervention group - Lower post-operative PROMIS pain intensity score in intervention group	N/A
Premkumar et al. [21]	2018	United States	Single-arm study	Orthopedics (total hip or knee arthroplasty)	183	N/A	Complication monitoring; pain monitoring and control	Daily information collection on patient-reported opioid consumption and pain	Text messages	- User engagement	- Overall response rate of 96.1%, defined as completion of at least 50% of twice-daily SMS post-operative questions for six weeks	N/A
Rojas et al. [22]	2019	United States	RCT	Orthopedics (musculoskeletal tumour patients)	14	N/A	Pain monitoring and control	CBT intervention text messages giving general post-operative guidance and encouragement	Text messages	- User engagement - Pain score - Patient-reported opioid use	- 90% completion rate of all questions in intervention group - Intervention group used less of daily prescribed opioid medication	N/A
Goz et al. [23]	2019	United States	Single-arm study	Orthopedics (spine surgery)	21	N/A	Patient education and instruction	Text messages to address common post-operative concerns of patients undergoing spine surgery	Text messages	- User satisfaction	- Average rating of the application on a 1 to 5 scale with 5 being “very useful” was 4.57	N/A
Wittig-Wells et al. [24]	2019	United States	RCT	Orthopedics (hip or knee arthroplasty)	29	N/A	Patient education and instruction	Daily pre-set cellular telephone alarm as a reminder for adults to take prescribed aspirin twice daily as antithrombotic therapy	Pre-set phone alarms	- Self-reported medication adherence	- Intervention group had lower rates of forgetting aspirin (29.7% vs. 59.5%)	N/A
van Dijk-Huisman et al. [25]	2019	The Netherlands	Non-randomized trial	Orthopedics (total hip or knee arthroplasty)	97	Hospital Fit	Functional recovery; patient education and instruction	Objective exercise monitoring with accelerometer and real-time feedback; provides personalized exercise programme	Smartphone app with accelerometer	- Time spent physically active - Functional recovery on POD1	- Intervention group patients stood and walked on POD1 for an average increase of 28.43 min over control - Odds of achieving functional recovery on POD1 was 3.08× higher	N/A
Belarmino et al. [26]	2019	United States	Single-arm study	Urology (robotic-assisted radical prostatectomy)	20	N/A	Patient education and instruction; functional recovery; pain monitoring and control	Peri-operative reminders to perform Kegel exercises, ambulate, and hydrate	IOS app	- User satisfaction	- 15 men (75%) who completed the satisfaction survey found the app easy to use and understand	N/A
Felbaum et al. [27]	2018	United States	Single-arm study	Neurosurgery (routine neurosurgery procedures)	56	TrackMyRecovery	Patient education and instruction; pain monitoring and control; complication monitoring; clear communication	Patient-specific pre- and post-operative instructions; pain scores and wound images can be reported through the app	iOS and Android app	- Successful registration and use of app - Compliance with reading instructions - Surgery cancellation - Post-operative complications within 6 weeks - Readmissions within 30 days - Number of peri-operative phone calls	- 54/56 patients successfully registered, downloaded, and complied with instructions both before and after surgery - There were no cancelled surgeries or readmissions. - There was 1 post-operative complication. - 8/54 patients called the office for a surgery related question.	N/A
Heuser et al. [28]	2021	Canada	Retrospective cohort study	Bariatric surgery (Roux-en-Y gastric bypass, sleeve gastrectomy)	854	N/A	Patient education and instruction; pain monitoring and control; complication monitoring	Notification reminders before surgery; peri-operative educational materials; post-operative symptoms and healthcare utilization monitoring	IOS and Android app	- Post-operative outcomes (LOS, ED visits, readmission) - Patient-reported self-care and healthcare utilization outcomes - User engagement - User satisfaction	- Use of the app was not associated with the rates of prolonged length of stay, ED visits, and readmission - 48.5% of surveyed patient reported the app helped them avoid phone calls to the hospital. 13.0% avoided ED visits - 66.2% of patients completed the daily health check survey at least once in the first week after the surgery - 94.8% of surveyed patients would recommend the app to other patients	N/A
van Hout et al. [29]	2022	The Netherlands	Single-arm study	General surgery (inguinal hernia repair)	242	Q1.6 Inguinal Hernia	Pain monitoring and control; complication monitoring	Continuous digital patient outcome measurement using non-intrusive, short questions	IOS and Android app	- Chronic post-operative inguinal pain - User compliance - Patient satisfaction - Other PROs (wound healing, pain, physical limitation, and potential recurrence)	- After months 3, 6, and 11, respectively, 3.0%, 4.4%, and 4.5% of patients reported inguinal pain or discomfort (NRS ≥ 4) - Compliance with the application was 91.7% after 14 days, 69.0% after 3 months, and 28.8% after 1 year - 92.8% of patients preferred the app over standard care	N/A
Sousa et al. [30]	2019	Brazil	Single-arm study	Maxillofacial surgery (orthognathic surgery)	30	OrtogApp	Patient education and instruction; clear communication	Provides five learning content sessions essential for managing peri-operative care; email contact with specialist nurses	IOS and Android app	- Usability - User satisfaction	- Satisfaction index was 82.9%	Average SUS of 79.8
Ashraf et al. [31]	2021	United Kingdom	Single-arm study	General surgery (colorectal surgery)	17	N/A	Patient education and instruction; complication monitoring	Three main components of education, physical, and psychological functional measurements (questionnaires and journal), and post-operative wound monitoring	iOS app	- Uptake and interaction - Usability	- 10/17 patients introduced to the app downloaded it and registered - Average number of interactions was 24 per patient - Interaction with the journal was the most frequent (33.7% of all usage)	N/A
Abdeen et al. [32]	2022	United States	Non-randomized trial	Orthopedics (total hip or knee arthroplasty)	274	N/A	Patient education and instruction	Customized messages including appointment reminders for pre-anesthetic appointment, links to educational videos, and real-time text message reminders to perform the requisite tasks of the peri-operative protocols	iOS and Android app	- Compliance to chlorhexidine gluconate shower and hydration protocol - LOS - Surgical site infection - 90-day readmission	- App users had increased adherence to the hydration protocol (OR = 3.17; *p* = 0.003) - App use was associated with shorter LOS - There was no difference in adherence to chlorhexidine gluconate, readmission, or surgical site infection	N/A
van der Meij et al. [33]	2018	The Netherlands	RCT	General surgery and gynecology (laparoscopic cholecystectomy, hernia inguinal surgery, or laparoscopic adnexal surgery)	344	ikHerstel	Functional recovery; patient education and instruction; clear communication	Recovery advice based on personalized convalescence plan; information about the peri-operative period; monitoring and feedback on recovery; e-consult feature	Smartphone app, website, and activity tracker	- Time elapsing between surgery and return to normal activities after surgery, measured by PROMIS physical function item	- Median time until return to normal activities was 21 days in the intervention group and 26 days in the control group (HR 1.38; *p* = 0.007).	N/A
Pickens et al. [34]	2019	United States	Single-arm study	General surgery (hepatectomy, distal pancreatectomy, pancreatico-duodenectomy)	122	N/A	Patient education and instruction; complication monitoring	Pre-operative scheduled task reminders for both general preparation; daily post-discharge health checks with customized responses to guide out-of-hospital care	Web-based platform	- User engagement - User satisfaction - PROs (post-operative pain, nausea, opiate use, compliance to ERAS^®^ pathway items) - Quality of life assessed by QoR-15 and PROMIS	- Application adoption was 93% and in-hospital engagement was 88% - Satisfaction rate was high with 86% recommending the application - Patients completed 62% of PROs - The 30-day end-of-study PROMIS survey was completed by 41% of participants	N/A

Acronyms: SUS: System Usability Scale; eHIQ: eHealth Impact Questionnaire; RCT: randomized controlled trial; ERAS^®^: Enhanced Recovery After Surgery; PROMIS: Patient-Reported Outcomes Measurement Information System; LOS: length of stay; SMS: short message service; CBT: cognitive behavioural therapy; POD: post-operative day; PRO: patient-reported outcomes; ED: emergency department; NRS: numeric rating scale; OR: odds ratio; HR: hazards ratio; QoR-15: Quality of Recovery 15 question survey.

**Table 2:** Peri-operative characteristics of an ideal peri-operative smartphone application.

**A.** **General characteristics should**	
	Be simple to download, easy to use, quick to learn, and not overly cumbersome or technically complex. Be designed to send out time-sensitive text messages directly to the patient rather than rely on the patient to open the application to view instructions or information. Reflect simple instructions that are easily interpreted. Be introduced early in the pre-operative period. Solidify the understanding of pre-operative counselling and prepare and guide patients optimally through the peri-operative period. Provide an interface that allows for interactions with surgical care providers. Have the option to allow for push notifications. Have a multimodal approach, including clear, concise videos or gamification that playfully encourage patients to acquire useful knowledge and engage in tasks. Provide advanced functions, such as personal logs with appointments and a more personalized prognosis. Provide clear expectations and guidelines that optimally guide and prepare patients for surgery and rehabilitation. Have multiple reminders for self-monitoring. Allow for user feedback and provide important avenues for improvement and guidance for future designs, such as real-time traffic data. Provide motivational features for increase in long-term user engagement. Allow for goal setting and self-reflection.
**B.** **Information and instructions provided by the application should**	
	Be clear and provided in a timely fashion. Be provided in a plain language style and refrain from technical terms. Incorporate information from existing pamphlets. Not be overly optimistic. Provide adequate reference data that is up to date. Have input and collaboration from experts, surgical care providers, and patients. Be aligned with information previously provided by the surgical care team. When appropriate, provide clear videos with exercises tailored to the condition, recovery, and functional tasks. Provide different types of media such as Photos of the operating room. Videos of post-operative rehabilitation exercises. Be ideally personalized and provide access to personal electronic health record. Provide reference data from peers. Be able to be used on a continuous timeline as opposed to jumping back to a previous phase of recovery instead of continuing with the current phase.

**Table 3:** Data metrics that should be included in the user attitude-based questionnaire.

**Questionnaires Should Include Data Metrics Such As**
- Does the application improve the overall surgical experience? - Does the application provide essential peri-operative reminders? - Does the application clarify information from the pre-operative appointment? - Would the patient like to have this application available during future surgery? - Was there any conflicting information relayed from the surgeon during counselling and the text messages sent by the application? - What is the patient’s attitude toward the information and presentation provided via the application? - Did the application improve the patient’s confidence in discussing health with others? - Did the application motivate the patient to manage their health? - What was the overall satisfaction with the application? - What were the most appreciated and used parts of the application? - What was the overall satisfaction with the amount of information provided? - Would the patient recommend the application to other patients? - To what extent did the patient feel that the application was supportive in addition to the information given by health professionals? - What were the strengths and limitations of the application? - Did the application provide patients with knowledge about their surgery and anesthesia, potential complications to monitor for, and the recovery process after? - What would the patient want to change about the application? - Why did the patient not use the mobile application?

## Data Availability

No new data were created or analyzed in this study. Data sharing is not applicable to this article.
